# Brain-Derived Neurotrophic Factor Polymorphism and Aphasia after Stroke

**DOI:** 10.1155/2021/8887012

**Published:** 2021-07-28

**Authors:** Nathan T. Lee, Fatimah Ahmedy, Natiara Mohamad Hashim, Khin Nyein Yin, Kai Ling Chin

**Affiliations:** ^1^Rehabilitation Medicine Unit, Faculty of Medicine & Health Sciences, Universiti Malaysia Sabah, Kota Kinabalu, Malaysia; ^2^Department of Rehabilitation Medicine, Faculty of Medicine, Universiti Teknologi MARA, Sg. Buloh, Malaysia; ^3^Department of Surgery, Faculty of Medicine & Health Sciences, Universiti Malaysia Sabah, Kota Kinabalu, Malaysia; ^4^Department of Biomedical Sciences, Faculty of Medicine & Health Sciences, Universiti Malaysia Sabah, Kota Kinabalu, Malaysia

## Abstract

Stroke is one of the most deliberating causes of mortality and disability worldwide. Studies have implicated *Val*66*Met* polymorphism of the brain-derived neurotrophic factor (BDNF) gene as a genetic factor influencing stroke recovery. Still, the role of BDNF polymorphism in poststroke aphasia is relatively unclear. This review assesses the recent evidence on the association between the BDNF polymorphism and aphasia recovery in poststroke patients. The article highlights BNDF polymorphism characteristics, speech and language interventions delivered, and the influence of BNDF polymorphism on poststroke aphasia recovery. We conducted a literature search through PubMed and Google Scholar with the following terms: “brain derived-neurotrophic factor” and “aphasia” for original articles from January 2000 until June 2020. Out of 69 search results, a detailed selection process produced a total of 3 articles that met the eligibility criteria. All three studies included *Val*66*Met* polymorphism as the studied human BDNF gene. One of the studies demonstrated insufficient evidence to conclude that BDNF polymorphism plays a role in poststroke aphasia recovery. The remaining two studies have shown that *Met* allele genotype (either single or double nucleotides) was associated with poor aphasia recovery, in either acute or chronic stroke. Carriers of the *Val*66*Met* polymorphism of BDNF gave a poorer response to aphasia intervention and presented with more severe aphasia.

## 1. Introduction

Stroke is one of the leading causes of death and acquired disability globally [1]. In addition, many demographic and clinical factors have influenced poststroke recovery, including age, stroke severity, presence of cognitive impairment, and neuropsychological deficits [2]. Thus, there is an emerging interest in studying genetic factors and variations that influence stroke susceptibility and recovery [3]. One genetic variation of interest is the *Val*66*Met* single-nucleotide polymorphism of the brain-derived neurotrophic factor (BDNF) gene in humans, a potential clinically significant genetic variation associated with stroke risk and prognosis [3]. The BDNF *Val*66*Met* polymorphism structurally involves the substitution of the amino acid valine (*Val*), to methionine (*Met*), in the 5′ ori-region of the human BDNF gene [4].

BDNF, being part of the neurotrophin family of growth factors, is believed to influence a wide range of aspects of the nervous system, including but not limited to neuronal migration, dendritic growth, synapse maintenance, and long-term plasticity [5]. However, the number of clinical research studies of the role of BDNF polymorphisms in stroke is limited, and the exact influence of BDNF polymorphisms underpinning the aspects of stroke severity, recovery, and functional outcome is still unclear [6].

The *Val*66*Met* of the BNDF gene, also known as rs6265, is only known to occur in humans and currently remains one of the most studied single-nucleotide polymorphisms of the BDNF gene [4]. In normal functioning, BDNF plays a significant neurological role in the modulation of hippocampal plasticity and hippocampal-dependent memory in humans and animals [4]. On the other hand, *Val*66*Met* mutation is associated with a reduction in the hippocampal tissue. Moreover, this mutation is linked hypothetically to several brain diseases, such as memory impairments and neuropsychiatric disorders [7].

The relationship between the language function and variations in the BDNF gene, however, is relatively less prominent. Nevertheless, evidence suggests that BDNF plays a significant role in learning and memory by inducing long-term potentiation (LTP), an essential form of synaptic plasticity [8]. A study by Winter et al. is aimed at investigating the effects of physical exercise and learning performance. They have demonstrated that the peripheral levels of BDNF were increased and sustained more strongly during learning (including language learning) after physical exercise in healthy adults. Here, it proved the role of BDNF as a mediator of exercise-induced learning improvement [9].

The main impetus of the present review is to investigate whether BDNF *Val*66*Met* polymorphism is associated with language function in people with poststroke aphasia. We hypothesized that the presence of the BDNF *Val*66*Met* polymorphism would affect the language function outcome after stroke.

## 2. Material and Methods

### 2.1. Search Methodology

Two reviewers conducted a literature search in PubMed and Google Scholar, with the following terms: “brain derived-neurotrophic factor” AND “aphasia” AND “stroke” for articles published from January 2011 to December 2020. The search results were then screened based on the subsequent inclusion and exclusion criteria. Any disagreements were resolved by consulting a third reviewer, if necessary.

### 2.2. Study Selection

The selected articles must be in English. We considered clinical studies that included BDNF evaluation as part of the main variables among adults with stroke (age 18 years and above). Case reports, review articles, technical reports, and thesis dissertation were excluded, as well as abstract-only publications. Studies that exclusively determined nonlanguage cognitive domains outcomes were also excluded.

### 2.3. Data Extraction and Recording

The following data were extracted and recorded: (i) details of article (title, author, year of publication, study design, and sample size); (ii) demographic and clinical characteristics of the studied population (mean age, type of stroke, and BDNF genotypes); and (iii) study outcomes (researched variables, interventions, outcome measures of assessment, and their corresponding results). Being a review article, the authors did not request ethical approval as the articles were already published.

## 3. Results

**Figure 1:**
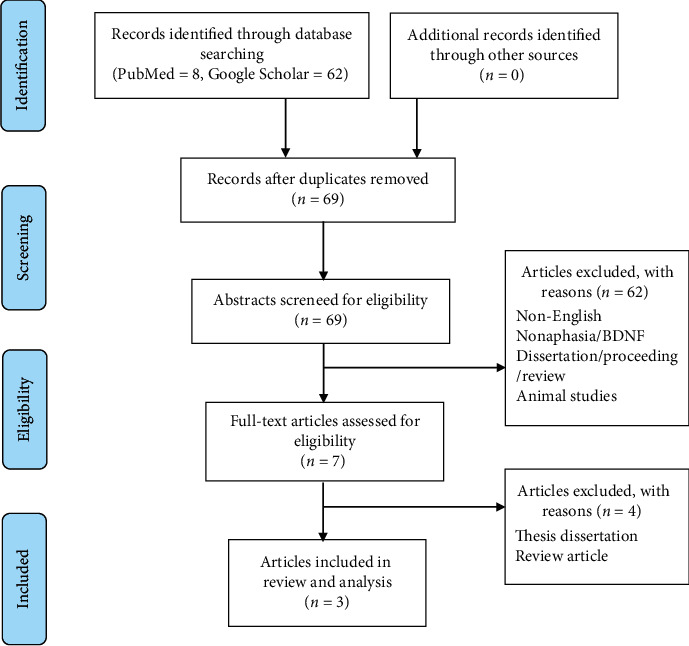
PRISM flowchart of the article selection process.

The electronic search resulted in 10 records. After removing duplicates, screening all the titles and abstracts, and accessing full articles of seven studies, a total of 3 articles were selected based on the eligibility criteria.  Figure 1 illustrates the PRISM flowchart on the selection process.  Table 1 summarizes the characteristics and key findings of the selected articles.

### 3.1. BDNF Polymorphism Characteristics

All three studies included *Val*66*Met* single-nucleotide polymorphism as the studied human BDNF gene [10–12]. de Boer et al. [10] carried out a prospective follow-up study to investigate the effects of the function limiting *Val*66*Met* polymorphism of BDNF on the recovery of poststroke aphasia in acute stroke. They divided the affected individuals into two groups based on their BDNF genotype, namely, carriers (with at least 1 Met allele) and noncarriers (absence of *Met* allele) [10]. A randomized controlled trial by Fridriksson et al. [11] investigated the response of different carriers of BDNF genotypes on behavioural aphasia treatment in acute stroke, while Kristinsson et al. [12] conducted a cross-sectional study to investigate how BDNF genotype may influence functional brain activation in chronic aphasia. Both divided the groupings of polymorphism into typical and atypical—the former is grouped based on having the *Val*66*Val* allele, i.e., BDNF polymorphism in the absence of *Met* allele. In contrast, the latter has at least one *Met* allele, either the Val66Met or Met66Met.

### 3.2. Speech and Language Intervention for Poststroke Aphasia

Only two studies investigated the effect of the intervention on language outcomes [10, 11]. All participants studied by de Boer et al. [10] received 2 to 5 hours of speech and language therapy (SLT) per week throughout the intake period of 2 years. The primary outcomes were measured using the Amsterdam-Nijmegen Everyday Language Tests (ANELT) and Boston Naming Test (BNT), assessed at baseline and discharge, in which both measures demonstrated improvement over time [10]. All acute stroke participants in the study by Fridriksson et al. [11] received 15 computerized language aphasia treatments, which focused on picture-word matching for 45 minutes, five times per week for three weeks. They were randomized to receive either 1 mA of anodal tDCS (transcranial direct stimulation current) or sham tDCS to the left temporoparietal region for the first 20 minutes of each session [11]. The therapeutic response to tDCS was assessed using the Philadelphia Naming Test (PNT), “Naming 80” test, and Western Aphasia Battery (WAB) test at one week, four weeks, and 24 weeks posttreatment [11].

### 3.3. Influences of BDNF Polymorphism on Poststroke Aphasia Recovery

Even though there were improvements in both ANELT and BNT, de Boer et al. [10] failed to demonstrate significant differences in both performances between the two groups at discharge, despite a large discrepancy in the baseline scores and the improvement scores across both groups. The study did not control confounding variables, including stroke severity, conditions for discharge, and social factors in this study [10]. The differences in the improvements of both the ANELT and BNT between both groups were not statistically significant [10]. The findings from this preliminary study suggested that there is insufficient evidence to conclude that BDNF polymorphism plays a role in poststroke aphasia recovery.

Based on the study by Fridriksson et al. [11], the baseline aphasia quotient (AQ) scores from revised Western Aphasia Battery (WAB) demonstrated that atypical BDNF genotype carriers had a more severe aphasia presentation than typical BDNF genotype carriers [11]. This result was consistent with the presumption that the atypical BDNF genotype leads to lower levels of BDNF secretion during activity [11]. Moreover, typical BDNF genotype patients exhibited improvement in naming for both A-tDCS and sham tDCS interventions [11]. Interestingly, contrary to the results from de Boer et al.'s study [10], Fridriksson et al. [11] demonstrated that the BDNF *Met* allele genotype has an impact on language performance and improvement in stroke. Furthermore, the latter showed that *Met* allele carriers of the BDNF gene produced a more unsatisfactory response to aphasia treatment than *Val*66*Val* and other typical genotype carriers of BDNF, regardless of the language therapy delivered [11]. In addition, Fridriksson et al. found no differences for other factors such as semantic processing, executive function, stroke severity, age, lesion size, education, or time poststroke between both groups [11].

Kristinsson et al. [12] used functional magnetic resonance imaging (MRI) for visualizing the cortical activation and WAB for measuring language impairment in two groups of participants with chronic stroke based on typical or atypical BNDF polymorphism carrier status [12]. First, the naming-related activation lesion contrast maps showed a relatively lesser activation present in the right hemisphere of the atypical group than the typical group [12]. Following this, they further quantified the MRI finding by obtaining the number of voxels present in predetermined regions of functional naming-related activated regions for each group of participants at the whole-brain level and both the left and right hemispheres, respectively and separately [12]. The typical genotype group demonstrated a higher number of activated voxels than the atypical group at both the whole-brain and right hemispheres [12].

In addition, participants in the atypical BDNF group had an overall greater aphasia severity on the revised-WAB-AQ than that of typical BDNF carriers of chronic stroke [12]. The findings of this study suggest that cortical brain activation is potentially mediated by BDNF genotypes, with reduced cortical activation of *Met* allele carriers [12]. There were no significant differences between both groups for baseline stroke severity, baseline aphasia severity, and executive functioning [12]. Age, racial distribution, education, lesion size, amount of exercise, or stroke severity differed between the two groups but reached nonsignificant levels [12].

## 4. Discussion

The majority of the results are primarily in line with established evidence when evaluating the impacts of BDNF polymorphism on poststroke outcomes [3, 6]. Typically, carriers of the *Met* allele of BDNF presented with poorer long-term functional outcomes after stroke [13, 14].

In addition, specific polymorphisms in the human BDNF gene are often linked to greater cognitive performance, including learning and memory, attention, and executive functions [4, 5]. Thus, it would be reasonable to assume that certain genetic variations in the BDNF gene are affiliated with language production and comprehension.

From the literature search performed, only three studies yielded the investigation of the correlation between language impairment or aphasia in stroke and BDNF genotypes, highly suggesting that knowledge in this topic of interest is relatively new and limited. Based on these findings, the *Val*66*Met* polymorphism of BDNF is linked with more severe aphasia at baseline [11, 12], poorer improvement in language improvement with time [11], and reduced cortical activation [12]. However, the exact role of BDNF polymorphisms in language performance and recovery in stroke may require further investigation.

The findings of de Boer et al. [10] demonstrated that the BDNF genotype is not specific to language performance and improvement, in contrast to the results of the other selected studies, which showed that BDNF genotypes are involved in the language outcome in stroke [13, 14]. Several possible explanations can be stipulated for such discrepancy. First, the study by de Boer et al. [10] received a relatively higher frequency of SLT compared to the intervention in [11]. In contrast, the study by Kristinsson et al. [12] did not account for the presence of SLT. Secondly, there were different standardized aphasia tests to assess aphasia: Dutch [10] and English [11, 12]. Here, the potential effects of bilingualism or multilingualism might require further investigation. Evidence suggests that bilingualism may be protective for adults with aphasia, possibly contributing to cognitive reserve in adults with aphasia [15]. Another consideration in these three prospective studies is the distinction between language recovery and language learning processes during stroke rehabilitation [10]. The significant variation in the improvement scores on the ANT and BNT further complicated the ability to detect significant differences between groups. Lastly, apraxia, which may affect the study results, was not excluded from the study [10].

Although the *Val*66*Met* allele of BDNF is associated with poorer language performance after tDCS intervention in poststroke aphasia [11], Marangolo et al. [16] demonstrated that the tDCS does not significantly alter the levels of BDNF on chronic aphasia patients. Thus, despite observing improvement in the scores of language performance, BDNF is not solely responsible for such improvement in language recovery after stroke. Fridriksson et al. [11] have hypothesized that this finding could be due to anodal tDCS (A-tDCS) dependence on baseline levels of BDNF secretion.

Contrary to the current theory that the *Met* allele of BDNF is linked with the defective intracellular secretion of BDNF, Lang et al. [17] have demonstrated that the *Val*66*Met* polymorphism of BDNF is associated with increased BDNF serum concentrations instead in healthy subjects. Furthermore, Lang et al. [17] postulated that the *Met* allele does not affect the constitutive secretion of BDNF but rather decreases the amount of activity-dependent BDNF secretion. Interestingly, Gajewski et al. [18] have shown that healthy elderly carriers of the Met allele of BDNF *Val*66*Met* outperformed homozygote (*Val*/*Val*) carriers of BDNF in task switching based on a cue-based and memory-based task. Their findings hypothesized that the *Met* allele contributes to more efficient cognitive processes under particular circumstances in healthy elderly subjects [18].

In addition, a study by Jasińska et al. [19] investigating the effects of BDNF *Val*66*Met* polymorphism on reading ability in children has shown that *Met* allele carriers of the BDNF gene experienced greater neural activation in the reading-related regions of the brain during a reading task. The performance of the *Met* allele carriers suggests that the BDNF polymorphism may be associated with phonological working memory, which is crucial in reading ability [19]. Moreover, Freundlieb et al. [20] have failed to find an association between BDNF *Val*66*Met* polymorphism and implicit short-term associative language learning paradigms in healthy adults.

Despite appreciating many established associations between variations of BDNF gene in stroke [3] with cognitive impairment and psychiatric disorders [7], the pondering question is whether BDNF can be considered a “disease susceptibility gene”. For stroke, BDNF *Val*66*Met* polymorphism is associated with long-term functional outcomes, with *Met* allele carriers exhibiting poorer modified Rankin scale scores [14, 15]. Nevertheless, there were weak associations between the BNDF gene and psychiatric conditions such as bipolar disorder [21]. Petryshen et al. [21] suggested that the variability in BDNF associations with psychiatric disorders could be attributed to the differences in population genetic structure. Hence, the diversity of BDNF polymorphism among worldwide populations would provide important implications for the implementation of further studies on poststroke aphasia. Furthermore, Kim et al. [13] have suggested that ethnic variability in the frequency of distribution of alleles may affect the positive findings to detect associations between BDNF genotypes and stroke outcomes.

Therefore, the BDNF gene may show a significant association with aphasia recovery after stroke, with the *Met* allele of the gene linked to poorer language recovery. In conclusion, some evidence suggests that polymorphism in the BDNF gene may modulate language recovery in poststroke aphasia. However, future research would be required to understand better the relationship between BDNF genetic variations and poststroke aphasia.

### 4.1. Study Limitations

The current review has several limitations. Firstly, there are a relatively limited number of original articles on the topic of BDNF polymorphism and poststroke aphasia-related outcomes, most prominently in the scope of BDNF genotypes. Hence, making strong inferences from a limited set of results concerning a topic as complex as the role of genetic polymorphism in aphasia would be challenging. In addition, there is variability in the parameters, interventions, and outcome measures utilized by the researchers. Finally, certain uncontrolled variables such as time of onset after stroke, type of aphasia, and presence and intensity of SLT have rendered the study populations as heterogeneous groups, which may have led to insufficient evidence for further statistical meta-analysis. These limitations justify future studies to explore the association between BDNF polymorphism and poststroke aphasia especially considering the emergence of neuromodulation therapy that promotes language improvement. In addition, establishing a more objective connection between these genotyping and the recovery of aphasia after stroke would enhance a better patients' selection for better utilization of resources.

## 5. Conclusion

There is some evidence suggesting that the *Met* allele of BDNF is associated with poorer language outcome in poststroke patients, in both acute and chronic stages. Further works are warranted to investigate this association to explore future treatments and strategies, which may produce therapeutic effects more efficiently for stroke patients. Employing an assumption that BDNF *Val*66*Met* polymorphism influences the severity and recovery of aphasia, identifying specific alleles of BDNF as a predictor for aphasia severity and recovery may be the next step targeting selective therapeutic strategies in stroke patients. However, our current understanding of the influence of specific genes in aphasia recovery is still relatively limited. Based on the findings of the selected articles, it seems that a correlation between BDNF polymorphism and aphasia recovery exists, although the exact mechanisms underpinning this effect are still unclear. Advancement in the study of the genetic influencers of aphasia may provide more efficient therapies for people with aphasia, therefore potentially improving the current prognosis of aphasia.

## Figures and Tables

**Table 1:** A summary of the included studies examining the effect of BDNF Val66Met single-nucleotide polymorphism on the recovery of poststroke aphasia.

Authors, year	Study design, sample size, mean age	Stroke type	BDNF genotypes	Intervention	Outcome assessment	Results/findings
de Boer et al., 2017 [10]	Prospective cohort study, 53 subjects, 58.5 years	Acute (ischemic and hemorrhagic)	(i) Val66Met allele present (ii) Val66Met allele absent	SLT for 2-5 hours per week	(1) ANELT (2) BNT	No significant differences between carriers of both alleles in improvement scores on both the ANELT and BNT
Fridriksson et al., 2018 [11]	Randomized controlled trial, 74 subjects (BDNF genotype available for 67), 61.7 years	Acute (ischemic and hemorrhagic)	(i) Atypical (Val/Met, Met/Met) (ii) Typical (Val/Val)	Received either 1 mA A-tDCS or sham tDCS for 20 mins per session for 5×/week for 3 weeks	(1) Naming 80 (2) PNT (3) WAB-R AQ	Atypical BDNF carriers showed significantly poorer response to A-tDCS than typical BDNF carriers who received both A-tDCS and S-tDCS Atypical BDNF carriers associated with poorer AQ scores at baseline compared to typical BDNF carriers
Kristinsson et al., 2019 [12]	Cross-sectional study, 87 subjects, 61.7 years	Chronic (ischemic and hemorrhagic)	(i) Atypical (Val66Met, Met66Met) (ii) Typical (Val66Val)	Not applicable	(1) WAB AQ (2) PNT (3) fMRI activation map analysis (4) Activated voxels at the whole-brain level	Atypical BDNF carriers significantly have more severe aphasia on WAB-AQ and performed significantly better in PNT compared to typical BDNF carriers No group differences between intensity of cortical activation across both groups The number of activated voxels was significantly lower in atypical BDNF carriers compared to typical BDNF carriers

BDNF: brain-derived neurotrophic factor; SLT: speech and language therapy; ANELT: Amsterdam-Nijmegen Everyday Language Test; BNT: Boston Naming Test; tDCS: transcranial direct current stimulation; A-tDCS: anodal tDCS; S-tDCS: sham tDCS; PNT: Philadelphia Naming Test; WAB: Western Aphasia Battery; WAB-R: Western Aphasia Battery, Revised; AQ: aphasia quotient; MRI: magnetic resonance imaging; fMRI: functional MRI.

## Data Availability

In view of the nature of the article, the authors do not own the data and all sources are cited appropriately.
